# The YmgB-SpoT interaction triggers the stringent response in *Escherichia coli*

**DOI:** 10.1016/j.jbc.2023.105429

**Published:** 2023-11-04

**Authors:** Paul Guiraud, Elsa Germain, Deborah Byrne, Etienne Maisonneuve

**Affiliations:** 1 Laboratoire de Chimie Bactérienne, Institut de Microbiologie de la Méditerranée, CNRS-Aix Marseille Univ (UMR7283), Marseille, France; 2 Protein Expression Facility, Institut de Microbiologie de la Méditerranée, CNRS-Aix Marseille Univ, Marseille, France

**Keywords:** (p)ppGpp, stringent response, SpoT, YmgB, AriR, bacterial stress response, *E. coli*

## Abstract

Virtually all bacterial species synthesize (p)ppGpp (guanosine penta- or tetraphosphate), a pleiotropic regulator of the so-called stringent response, which controls many aspects of cellular physiology and metabolism. In *Escherichia coli*, (p)ppGpp levels are controlled by two homologous enzymes: the (p)ppGpp synthetase RelA and the bifunctional synthetase/hydrolase SpoT. We recently identified several protein candidates that can modulate (p)ppGpp levels in *E. coli.* In this work, we show that the putative two-component system connector protein YmgB can promote SpoT-dependent accumulation of ppGpp in *E. coli.* Importantly, we determined that the control of SpoT activities by YmgB is independent of its proposed role in the two-component Rcs system, and these two functions can be uncoupled. Using genetic and structure–function analysis, we show that the regulation of SpoT activities by YmgB occurs by functional and direct binding *in vivo* and *in vitro* to the TGS and Helical domains of SpoT. These results further support the role of these domains in controlling the reciprocal enzymatic states.

To survive, all living cells have developed robust and adaptive responses to rapidly cope with the damaging, potentially lethal, effects of environmental stresses. Virtually all bacteria produce the intracellular signaling molecules guanosine 5′-diphosphate 3′-diphosphate (ppGpp) and guanosine 5′-triphosphate 3′-diphosphate (pppGpp) in response to nutritional and environmental stresses. These alarmones, commonly known as (p)ppGpp, are secondary messengers that rewire cellular metabolism by triggering physiological changes allowing bacteria to adapt and survive nutritional and environmental stresses. Remarkably, this profound change in cell physiology also appears to play a key role in virulence, immune evasion, and antibiotic tolerance (1, 2, 3, 4).

The RelA-SpoT Homolog (RSH) family is responsible for the control of (p)ppGpp intracellular levels by synthesizing and degrading (p)ppGpp (5). Long RSH enzymes share similar domain organization. The enzymatic N-terminal region contains the synthetase and hydrolase domains, and the C-terminal region contains conserved domains that play a central role in sensing and transducing stress signals to the catalytic domains (6, 7, 8, 9). Therefore, the synthetase and hydrolase domains work in concert and the switch between the two enzymatic activities determines (p)ppGpp levels depending on the environmental cues. *Escherichia coli* possesses two long RSH paralogs called RelA and SpoT that emerged from gene duplication (5). Both RelA and SpoT can synthesize (p)ppGpp from GTP and GDP using ATP as a donor of the pyrophosphate moiety. However, despite strong sequence homologies and similar domain architecture, SpoT but not RelA can hydrolyze (p)ppGpp. RelA synthetic activity is required under amino acid starvation. This condition leads to a complex formation between RelA and deacylated tRNA. The loading of this complex at the ribosomal A-site activates RelA (p)ppGpp synthetic activity (10, 11, 12, 13).

In contrast, SpoT is a weak (p)ppGpp synthetase responsive to various environmental signals such as fatty acid (14), carbon (15), iron (16), and phosphate starvation (17). In addition, the hydrolytic activity of SpoT is essential for balancing intracellular (p)ppGpp concentration in the presence of RelA and disruption of *spoT* in wild-type *E. coli* strain is lethal (15). Therefore, the control of (p)ppGpp levels by SpoT represents an obvious checkpoint with implications that may result in changes in either synthetase or hydrolase activity to ensure rapid adaptation.

Importantly, heterologous protein interaction has been shown to play a critical role in the control of the reciprocal SpoT activities. Indeed, SpoT interacts with the acyl carrier protein (ACP), a central cofactor of fatty acid synthesis. This direct interaction between the TGS domain (Thr-tRNA synthetase, GTPase, and SpoT domain) in the C-terminal half of SpoT and the Holo form of ACP is required for (p)ppGpp accumulation in response to fatty acid limitation (18, 19). Similar to ACP, the anti-σ70 factor Rsd also interacts with the TGS domain of SpoT to stimulate the hydrolase activity upon carbon downshift (20).

More recently, to gain further insights into the regulation of SpoT activities, we have developed a genetic assay that allowed us to identify several protein candidates that can modulate SpoT-dependent (p)ppGpp accumulation in *E. coli* (21). Analysis of the first candidate, YtfK, revealed that it is required to maintain elevated ppGpp levels in response to phosphate and fatty acid starvation, therefore ensuring cell survival. This regulation occurs by a direct binding between YtfK and the catalytic domains of SpoT (21).

Here, we present the characterization of a second protein candidate, YmgB (also known as AriR), previously proposed to act as a connector of the Rcs phosphorelay (22) and involved in biofilm formation and acid resistance (23). We show that YmgB can trigger the accumulation of ppGpp. Moreover, this accumulation is independent of the previously observed connection between YmgB and the two-component system Rcs. Rather, we show that YmgB can promote ppGpp accumulation in a SpoT-dependent manner through a specific and functional interaction with the TGS and helical domains in the C-terminal regulatory region of SpoT.

## Results

### Overexpression of *ymgB* stimulates SpoT-dependent ppGpp accumulation

We previously presented a genetic screening assay that allowed us to identify novel proteins that can modulate SpoT-dependent accumulation of (p)ppGpp in *E. coli* (21). By using a plasmid pool from the ASKA library (containing almost all *E. coli* K-12 genes each cloned into high-copy-number plasmid), we selected for genes that, in multiple copies, suppressed the growth defect of a Δ*relA* strain in presence of 1 mM Serine, Methionine, and Glycine (SMG). These conditions induced isoleucine starvation and required elevated levels of (p)ppGpp to enable bacterial growth (24). Among the candidate genes, we isolated *ymgB* (also known as *ariR*) coding for a small protein (21).

**Figure 1:**
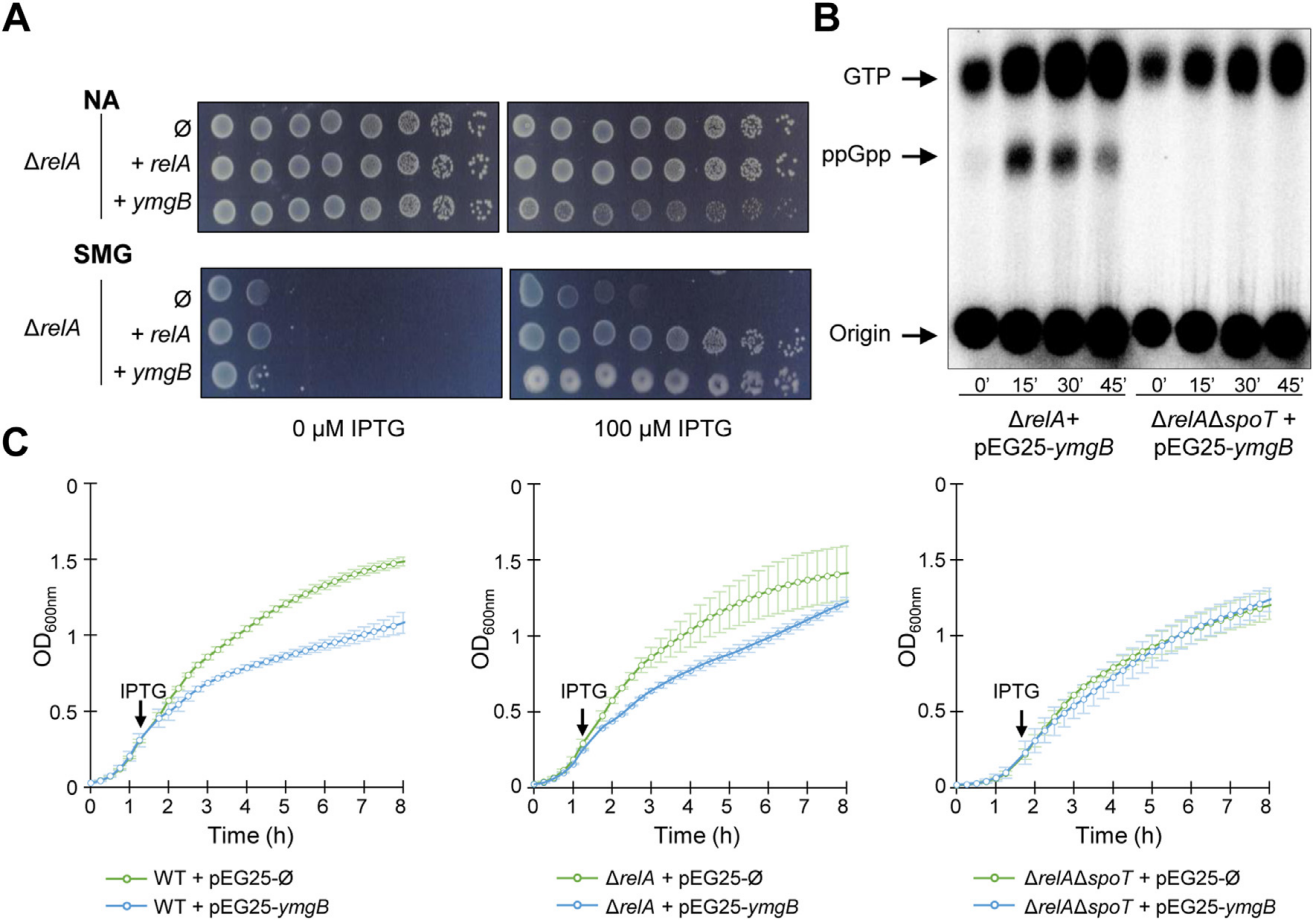
**YmgB stimulates SpoT-dependent ppGpp accumulation**. *A*, *ymgB* overexpression suppresses the non-growing phenotype of the Δ*relA* mutant on SMG plates. The Δ*relA* mutant was transformed with pEG25 (Ø) or pEG25 harboring either *relA* or *ymgB* under an IPTG-inducible promoter. Serial dilutions of stationary-phase cultures were spotted on nutrient agar (NA) and SMG plates with 0 or 100 μM IPTG. The results are representative of three independent experiments with similar results. Additional strains and IPTG concentrations are presented in Fig. S1*A*. *B*, *in vivo* (p)ppGpp accumulation observed after ectopic expression of *ymgB*. The *ΔrelA* and the (p)ppGpp^0^ (*ΔrelA ΔspoT*) strains carrying *ymgB* on pEG25 were grown exponentially in phosphate MOPS minimal medium as described in the Experimental procedures section. Samples were collected before and after *ymgB* induction (1 mM IPTG) prior to nucleotide extraction and separation by TLC. A representative autoradiogram of the TLC plates is shown. This experiment was repeated three times with a similar pattern of (p)ppGpp accumulation. *C*, growth curve of WT (*left panel*), Δ*relA* (*center panel*), and Δ*relA* Δ*spoT* strain (*right panel*) transformed with empty pEG25 (Ø) (*green lane*) or with pEG25 harboring *ymgB* (*blue lan*e) in LB medium. The overnight culture was 100 times diluted and growth was monitored at 600 nm by using a TECAN microplate reader. At the indicated time, 200 μM of IPTG was added to induce *ymgB* expression. Error bars indicate the standard deviations of averages of three independent experiments.

To further characterize this new candidate, we first confirmed the result obtained with the high-copy-number vector derivative pCA24N from the ASKA library after re-cloning the coding region of *ymgB* into a more suitable plasmid for physiological analysis harboring a tightly controlled, IPTG-inducible Pt5-Lac promoter (pEG25). As shown in Figures 1*A* and S1, *A* and *B*, YmgB repetitively suppresses the growth defect of a Δ*relA* mutant on SMG medium. To assess the role of YmgB in the stringent response and (p)ppGpp homeostasis, we monitored the (p)ppGpp level *in vivo* after ectopic *ymgB* overexpression in a Δ*relA* strain. As shown in Figure 1*B*, the ppGpp level sharply increased 15 min after *ymgB* induction.

Finally, we ensured that YmgB is not a small alarmone ppGpp synthetase. Indeed, induction of *ymgB* does not suppress the well-known amino acid auxotrophic phenotype of the ppGpp^0^ (Fig. S1*C*) strain (Δ*relA* Δ*spoT* mutant) and failed to induce ppGpp accumulation (Fig. 1*B*). Moreover, and consistent with the observation that at high concentrations ppGpp becomes a potent inhibitor of bacterial cell growth, we observed that induction of *ymgB* in rich medium (with an IPTG concentration above 200 μM) strongly impairs cell growth in both WT and Δ*relA* strains but not in the ppGpp^0^ strain (Figs. 1*C* and S1, *A* and *C*).

Taken together, our results show that YmgB can trigger a SpoT-dependent accumulation of ppGpp in *E. coli*.

### The control of SpoT activities by YmgB can be uncoupled from its role in the Rcs system

**Figure 2:**
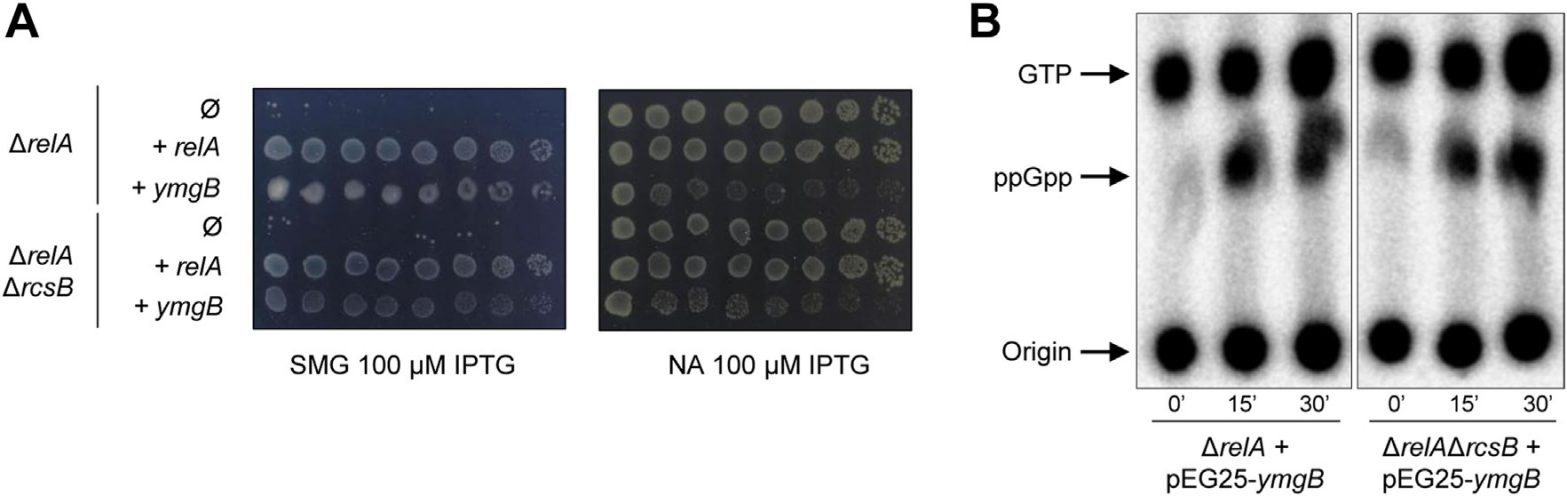
**SpoT-dependent accumulation of ppGpp is independent of Rcs phosphorelay**. *A*, the Δ*relA* and Δ*relA* Δ*rcsB* strains were transformed with pEG25 (Ø) or pEG25 harboring *relA* or *ymgB* under an IPTG-inducible promoter. Serial dilutions of stationary-phase cultures were spotted on NA and SMG plates supplemented with 100 μM IPTG. Additional strains and IPTG concentrations are presented in Fig. S2. This experiment was repeated three times with identical results. *B*, *in vivo* (p)ppGpp accumulation observed after ectopic expression of *ymgB*. The Δ*relA* and the Δ*relA* Δ*rcsB* mutants carrying *ymgB* on pEG25 were grown exponentially in phosphate MOPS minimal medium as described in the Experimental procedures section. Samples were collected before and after *ymgB* induction (1 mM IPTG) prior to nucleotide extraction and separation by TLC. A representative autoradiogram of the TLC plates is shown. This experiment was repeated three times with a similar pattern of (p)ppGpp accumulation.

YmgB is a small protein of 88 amino acids encoded by the *ycgZ-ymgABC* operon. YmgB has been previously proposed to act as a connector of the phosphorelay Rcs involved in biofilm formation and acidic stress resistance (22, 23). Therefore, to address if the observed stimulatory effect on SpoT-dependent ppGpp accumulation is dependent on its described function on the Rcs system, we used strains deleted for the Rcs response regulator RcsB (Δ*rcsB* and Δ*relA* Δ*rcsB* strains). The results presented in Figures 2 and S2 show that *ymgB* overexpression still suppresses the Δ*relA* Δ*rcsB* growth defect on SMG plates and induces ppGpp accumulation similar to what is observed in the *ΔrelA* strain. These results clearly demonstrate that activation of SpoT-dependent ppGpp accumulation is independent of the Rcs system.

Interestingly, we observed that *ymgB* ectopic expression induces a mucoid phenotype in WT, Δ*relA*, and Δ*relA* Δ*spoT* strains on SMG plate (Figs. 1, 2, S1, and S2). This mucoid phenotype is due to capsule synthesis, which is under the control of the Rcs system (22, 25, 26). Importantly, *ymgB* overexpression suppresses the growth defect on SMG but does not induce mucoid phenotype in the Δ*rcsB* and Δ*relA* Δ*rcsB* strains. Moreover, we observed that *ymgB* overexpression in the Δ*relA* Δ*spoT* strain induces formation of mucoid phenotype when grown on nutrient agar (NA) plates (Fig. S1*C*).

Taken together, our results show that YmgB acts independently on the Rcs system and on SpoT activities.

### The SpoT-YmgB ratio appears to control the switch of SpoT activities

**Figure 3:**
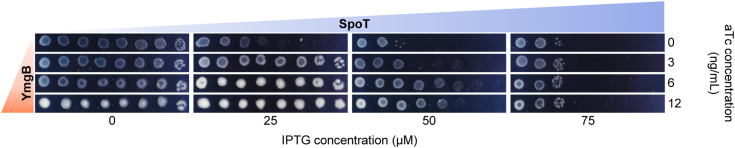
**The YmgB/SpoT ratio controls growth on SMG plates.** The WT strain was co-transformed with pEG25 harboring *spoT* under an IPTG-inducible promoter and with pBbS2K harboring *ymgB* gene under an anhydrotetracycline (aTc) promoter. Serial dilutions of stationary-phase cultures were spotted on SMG and NA medium with a gradual concentration of IPTG (to induce *spoT*) and aTc (to induce *ymgB*). Experiments have been repeated three times with similar results. Additional controls are provided in Fig. S3.

Our observation that ectopic induction of YmgB can promote ppGpp accumulation even in absence of nutritional stress suggests that YmgB protein levels play an important role in adjusting the level of intracellular ppGpp and that the regulation of SpoT activity by YmgB occurs through a change in the ratio between these two proteins *in vivo*. To address this assumption, we followed the growth phenotype of the WT strain on SMG plates as a function of SpoT and YmgB levels in a double expression system assay. As mentioned above growth on SMG plates requires a high (p)ppGpp level and can therefore be used as an indicator of SpoT activities. The gradual induction of SpoT progressively affected WT growth on SMG plate, consistent with a reduced basal level of (p)ppGpp as previously observed (21, 27). These results confirm that the artificial increase of SpoT level controls the switching from ppGpp synthesis toward degradation (21, 27) (Figs. 3 and S3). However, we observed that this growth defect requires higher concentrations of SpoT when YmgB level is increased (Figs. 3 and S3). These results suggest that switching from ppGpp degradation to synthesis can be directly determined by the SpoT-YmgB ratio and does not rely on additional input. Taken together our results show that the synthetase activity of SpoT is subjected to YmgB limitation *in vivo*.

### The YmgB-SpoT interaction promotes ppGpp accumulation

Several proteins have been previously reported to interact with SpoT to modulate the switch between the two antagonistic activities (18, 19, 20, 21, 28).

**Figure 4:**
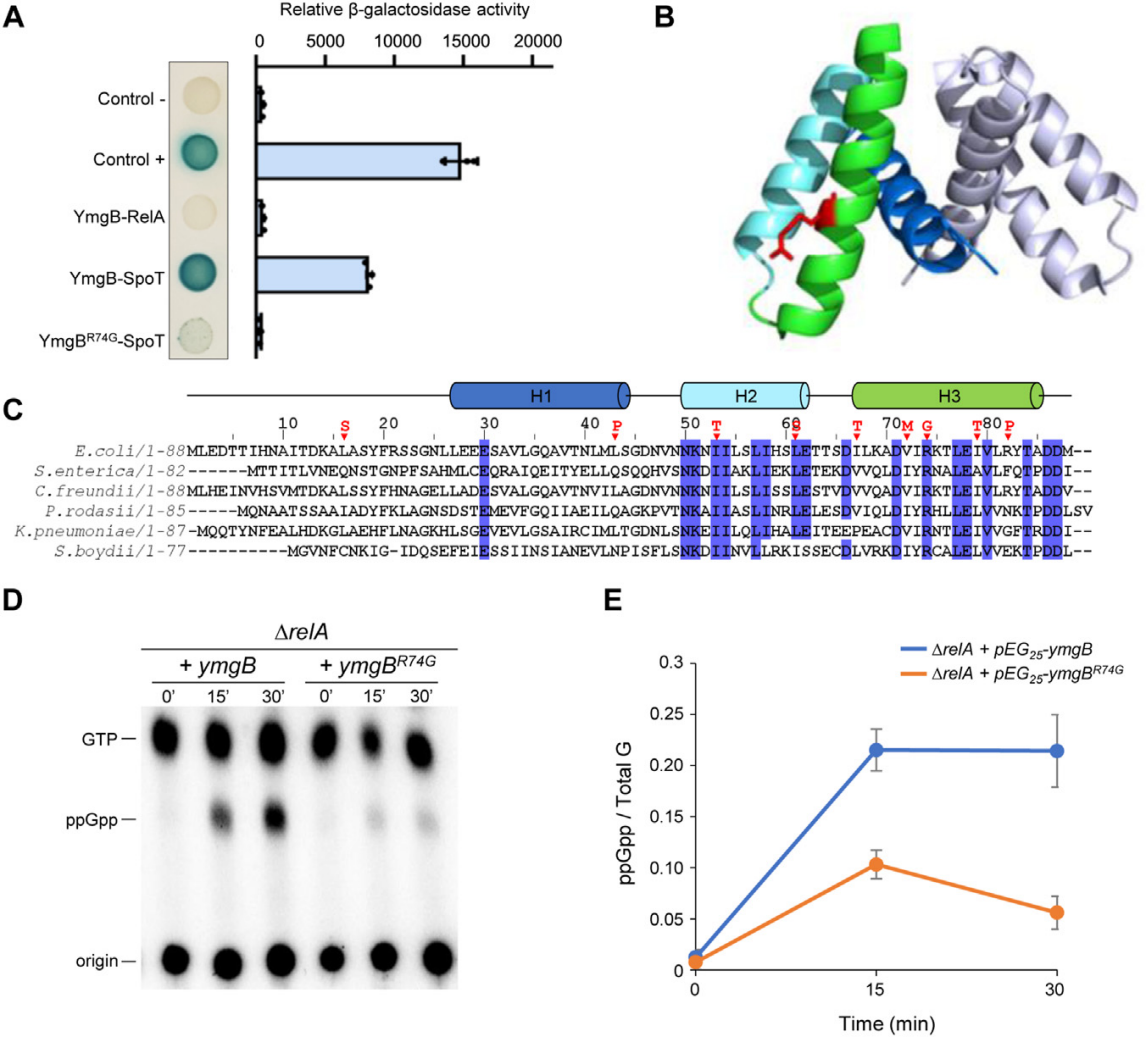
**The YmgB-SpoT interaction promotes ppGpp accumulation**. *A*, YmgB interacts with SpoT in BTH assay. Overnight cultures of BTH101 strains harboring pUT18c-*ymgB* (or pUT18c-*ymgB*^*R74G*^) and pKT25-*spoT* (or pKT25-*relA*) were spotted on X-Gal NA plates (see the Experimental procedures section). The *blue* color indicates a positive interaction. The results of the β-galactosidase assays using the same strains are shown on the horizontal graphs on the right. Error bars indicate the SDs of the means of three independent experiments. *B*, representation of YmgB head-to-head dimerization obtained by X-ray crystallography (2OXL) (23). The conserved R74 residue is presented as a red stick. *C*, multiple sequence alignment of six YmgB homologous across Enterobacteriaceae family with Kalign (3.3.1) program. Residues conserved for more than 80% of these six homologs are highlighted in *blue*. The residue positions affected by the 9 simple substitutions causing the loss of the YmgB-SpoT interaction are shown (▼). *D*, *in vivo* (p)ppGpp accumulation following ectopic expression of *ymgB* and *ymgB*^*R74G*^. The Δ*relA* mutant carrying either *ymgB* or *ymgB*^*R74G*^ on pEG25 was grown exponentially in phosphate MOPS minimal medium (see Experimental procedures section). Samples were collected before and after *ymgB* induction (1 mM IPTG) prior to nucleotide extraction and separation by TLC. The autoradiogram is representative of three independent experiments and the curves of the relative levels of ppGpp (*E*) are represented as the means of the three independent experiments, the error bars depict the SDs.

We therefore tested whether SpoT is able to physically interact with YmgB *in vivo* using a bacterial two-hybrid (BTH) assay (29). For that purpose, the complementary T18 and T25 domains of *Bordetella pertussis* adenylate cyclase were fused respectively to the N-terminus of YmgB and SpoT proteins. Plasmids encoding these fusions were used to co-transform the *cya*-deficient *E. coli* strain BTH101. As shown in Figures 4*A* and S4, *A* and *B*, YmgB exhibited a strong interaction *in vivo* with SpoT. However, despite the strong sequence homologies between SpoT and RelA, no interaction is observed between YmgB and RelA *in vivo* by BTH assay (Fig. 4*A*). To further assess the importance of this interaction, we randomly mutagenized YmgB and isolated nine independent single amino acid substitutions showing reduced interaction with SpoT (see Experimental procedures). As shown in Figure 4, *B* and *C*, the majority of these substitutions are located in the third alpha helix. One of these mutants that had a conserved surface exposed Arginine 74 substituted with Glycine (R74G) displayed an abolished interaction with SpoT (Fig. 4, *A* and *B*).

Moreover, we confirm that both T18-YmgB and T18-YmgB^R74G^ recombinant proteins were correctly produced, as shown by their ability do dimerize *in vivo* by BTH (Fig. S4*A*) (23). Importantly, induction of YmgB^R74G^ fails to suppress the growth defect of the Δ*relA* mutant on SMG plates (Fig. S4*C*). This result suggests that breaking the interaction with SpoT abolishes the stimulatory effect of YmgB on ppGpp accumulation. Indeed, even if YmgB and YmgB^R74G^ are produced at similar levels, induction of YmgB^R74G^ does not promote ppGpp accumulation when overproduced as compared to the strong accumulation when the WT copy of *ymgB* is expressed (Figs. 4, *D* and *E* and S4, *D* and *E*). Finally, we observed that ectopic expression of *ymgB*^*R74G*^ induces a mucoid phenotype similar to what is observed when the WT copy of *ymgB* is expressed showing that this variant is still able to activate the Rcs phosphorelay (Fig. S4*F*).

Taken together, our results show that a specific and functional interaction between SpoT and YmgB controls the antagonistic activities of SpoT to promote ppGpp accumulation *in vivo*.

### Suppressive mutation on SpoT restores the interaction with YmgB^R74G^ and ppGpp accumulation

**Figure 5:**
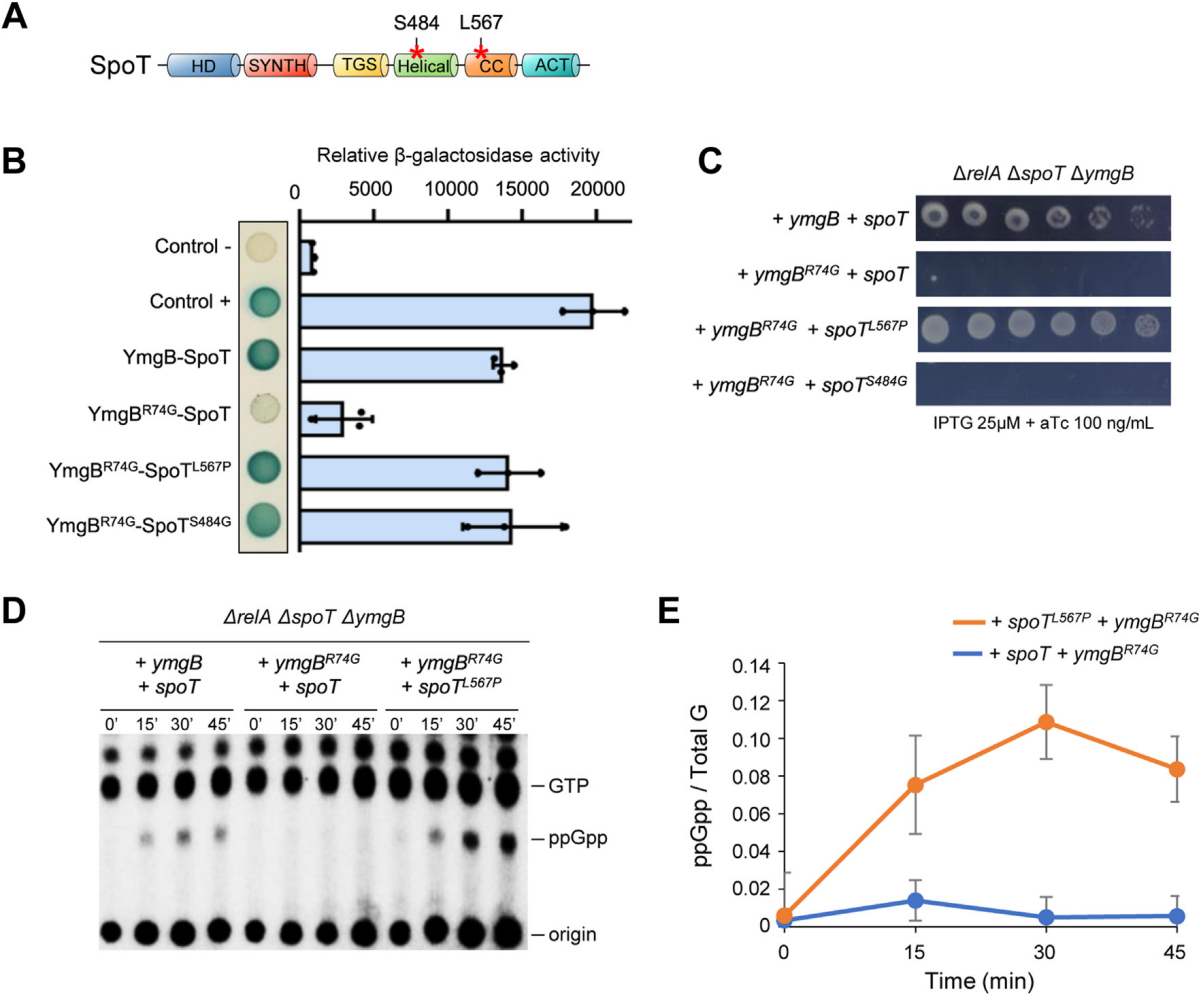
**Suppressive mutations on *spoT* restore the interaction with YmgB**^**R74G**^**and SpoT-dependent ppGpp accumulation**. *A*, schematic representation of SpoT domains organization. SpoT consists of a synthetase (SYNTH) domain, a hydrolase (HD) domain, a Thr-tRNA synthetase, GTPase and SpoT (TGS) domain, a Helical domain, a domain containing conserved cysteines (CC), and an Aspartokinase, Chorismate mutase, and TyrA (ACT) domain. The two single amino acid substitutions that independently restored interaction with YmgB^R74G^-SpoT are indicated (∗). One of such mutants had an amino acid substitution located in the Helical domain (Serine 484 to Glycine) and the other had a substitution located in the CC domain (Lysine 567 to Proline). *B*, the amino acid substitutions L567P and S484G on SpoT allow interaction with YmgB^R74G^ in BTH assays. Overnight cultures of BTH101 strains harboring pUT18c-*ymgB* (or pUT18c-*ymgB*^*R74G*^) and pKT25-*spoT*, pKT25-*spoT*^*L567P*^ or pKT25-*spoT*^*S484G*^ were spotted on X-Gal NA plates. The blue color indicates a positive interaction. The results of the β-galactosidase assays using the same strains are shown on the horizontal graphs on the right. Error bars indicate the SDs of the means of three independent experiments. *C*, *spoT*^*L567P*^ is able to suppress the growth of the Δ*relA* mutant on SMG plates when co-expressed with *ymgB*^*R74G*^. The Δ*relA* Δ*spoT* Δ*ymgB* strain was co-transformed with pEG25 harboring *spoT*, *spoT*^*L567P*^ or *spoT*^*S484G*^ gene under an IPTG-inducible promoter and with pBbS2K harboring *ymgB* or *ymgB*^*R74G*^ gene under an anhydrotetracyline (aTc) promoter. Cells were serially diluted and spotted on SMG medium with 25 μM of IPTG (to induce *spoT*) and 100 ng/ml of aTc (to induce *ymgB*). Additional controls are provided in Fig. S5*A*. Experiments have been repeated three times with similar results. *D*, *in vivo* (p)ppGpp accumulation during ectopic expression of *ymgB* (or *ymgB*^*R74G*^) and *spoT* (or *spoT*^*L657P*^) in a Δ*relA* Δ*spoT* Δ*ymgB* strain. The strains were grown exponentially in phosphate MOPS minimal medium with 5 μM IPTG. Samples were collected before and after *ymgB* induction (200 ng/ml aTc) prior to nucleotide extraction and separation by TLC. The autoradiogram is representative of three independent experiments and the curves of the relative levels of ppGpp (*E*) are represented as the means of the three independent experiments, the error bars depict the SDs. Additional control is provided in Fig. S5*B*.

To further unveil the functional relevance of the YmgB-SpoT interaction and its role in ppGpp accumulation, we randomly mutagenized SpoT and used the BTH assay to identify mutations that re-established the interaction with YmgB^R74G^. This approach generated two single amino acid substitutions in SpoT that independently fully restored the YmgB^R74G^-SpoT interaction signal (Fig. 5, *A* and *B*). We next used a (p)ppGpp^0^ strain to assess the functionality of these restored interactions. Remarkably, co-expression of the *spoT*^*L567P*^ and *ymgB*^*R74G*^ variants suppresses the growth defect of a (p)ppGpp^0^ strain on SMG plate (Figs. 5*C* and S5*A*). This result predicts that restoring the interaction between the two variants restores ppGpp accumulation. Indeed, as judged by TLC analysis, the consecutive expression of *spoT*^*L567P*^ and *ymgB*^*R74G*^ is associated with a rapid induction of ppGpp similar to that observed when the WT copies of these alleles are co-expressed (Figs. 5, *D* and *E* and S5*B*). Finally, we observed that while SpoT^S^^4^^84G^ restores the interaction with YmgB^R74G^, the expression of these alleles is not sufficient to suppress the growth defect of a (p)ppGpp^0^ strain on SMG plate. However, co-expression of *ymgB* and *spoT*^*S*^^*4*^^*84G*^ suppresses the growth defect on SMG plate, showing that SpoT^S^^4^^84G^ is functional (Fig. S5*A*). These observations support that a specific and functional interaction between YmgB and SpoT is needed to promote ppGpp accumulation. Taken together, our results further support the relevance and the functionality of the YmgB-SpoT interaction in controlling ppGpp homeostasis. Finally, identification of an allele-specific suppression is interpreted as the first evidence of a direct physical interaction between YmgB and SpoT.

### YmgB targets the C-terminal regulatory region of SpoT

**Figure 6:**
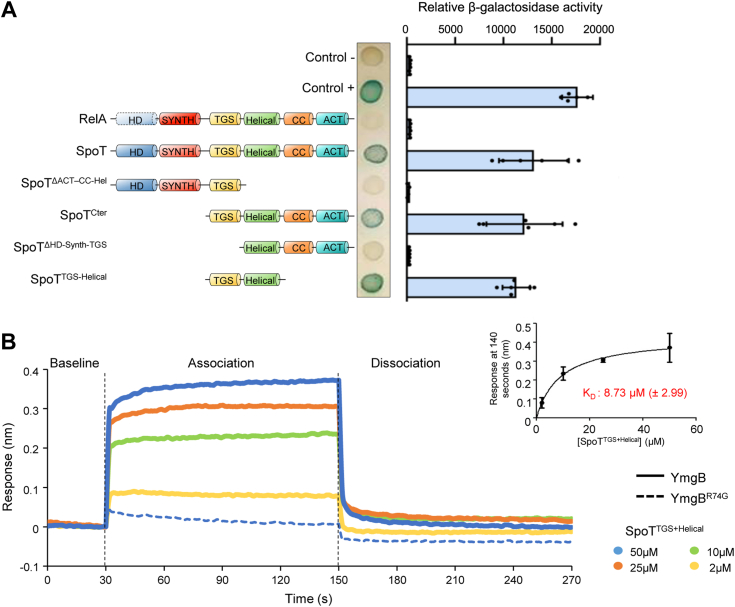
**YmgB interacts directly with the TGS and Helical domains of SpoT.***A*, the TGS and Helical domains of SpoT are sufficient to interact with YmgB in BTH assays. *E. coli* BTH101 cells were co-transformed with plasmid derivatives pUT18c-*ymgB* and pKT25 with the full-length or truncated *spoT* gene as indicated. Stationary-phase cultures were spotted on NA plates containing X-Gal as a blue color reporter for positive interaction. The bars showing β-galactosidase activity are represented as the means of three independent experiments, the error bars depict the SDs. Additional constructions are presented in Fig. S6*A*. *B*, YmgB can interact with SpoT *in vitro* by BioLayer Interferometry assay (BLI). Biotinylated YmgB or YmgB^R74G^ were immobilized on streptavidin biosensors and probed with SpoT^TGS-Helical^ at concentrations ranging from 2 to 50 μM. The curves are represented as the means of the subtracted reference binding responses during association and dissociation from three experiments. The inset curve shows the specific BLI response (nm) 10 s before the end of association as a function of SpoT^TGS-helical^ concentration. Data are represented as the means of the three experiments, the error bars depicts the SDs. A predicted structure of theYmgB-SpoT complex is presented in Fig. S7.

To better understand how YmgB controls SpoT activities, we determined which domains of SpoT are involved in the interaction with YmgB. SpoT consists of two functional regions, the N-terminal half comprising the synthetase (SYNTH) and hydrolase (HD) domains, and the C-terminal regulatory part encompassing a Thr-tRNA synthetase, GTPase and SpoT (TGS) domain, a Helical domain, a domain containing conserved cysteines (CC), and an Aspartokinase, Chorismate mutase and TyrA (ACT) domain. We therefore constructed truncated SpoT proteins fused to the T25 domain of *B. pertussis* adenylate cyclase and performed BTH analysis (Figs. 6*A* and S6*A*). This analysis first revealed that YmgB interacts with the SpoT^C-terminal^ fusion *in vivo* (Fig. 6*A*). Moreover, SpoT protein fusions lacking either the TGS or the helical domain fail to interact with YmgB (Fig. 6*A*). Finally, the TGS-Helical fusion continues to interact with YmgB but not with YmgB^R74G^, showing that these domains are necessary and sufficient for the SpoT-YmgB interaction (Figs. 6*A* and S4*A*).

We next used BioLayer interferometry (BLI), an *in vitro* protein-protein interaction method to confirm the interaction of YmgB at the TGS-Helical domains in the C-terminal region of SpoT observed *in vivo*. YmgB, YmgB^R74G^ and the SpoT^TGS-Helical^ domains were therefore purified by two consecutive chromatography experiments, affinity and size-exclusion (Fig. S6, *B*–*D*). Biotinylated YmgB or YmgB^R74G^ were immobilized on streptavidin biosensors and used as ligand. Interaction experiments were performed with purified SpoT^TGS-Helical^ as the analyte. Upon addition of SpoT, at concentrations ranging from 2 to 50 μM, a dose-response association is observed followed by a decrease during the dissociation step corresponding to the washing of the biosensor (Fig. 6*B*). These results show that YmgB directly interacts *in vitro* with the TGS-Helical domains of SpoT with a calculated dissociation constant (K_D_) of 8.73 μM (±2.99 μM) (Fig. 6*B*). Finally, and in agreement with the BTH assays, SpoT^TGS-Helical^ protein does not interact with the YmgB^R74G^ variant *in vitro*, further supporting the strong specificity of the interaction between YmgB and SpoT (Fig. 6*B*).

Collectively, our results show that the TGS-Helical domains in the C-terminal regulatory part of SpoT are necessary and sufficient to interact with YmgB both *in vivo* and *in vitro*.

## Discussion

Cells must constantly adjust the synthesis of (p)ppGpp with its hydrolysis to maintain an optimal cellular concentration of these alarmones in response to their environment. The intracellular pool of (p)ppGpp is mainly governed by the widely conserved protein family RSH. Over the past decade, diverse molecular mechanisms regulating the activity of RSH have been characterized (30, 31, 32, 33). *E. coli* contains two long RSH enzymes, RelA and SpoT, that synthesize the alarmones (p)ppGpp. While SpoT is also able to efficiently hydrolyze (p)ppGpp, RelA has a non-functional relic hydrolase domain, making SpoT the primary source of hydrolysis (15).

In this study, we expand the repertoire of how diverse protein partners interact with RSH proteins to control their enzymatic activities and provide an additional control point in the complex regulatory network underlying the regulation of the stringent response in *E. coli*. Indeed, we reported an additional mode of regulation that can promote intracellular ppGpp accumulation in *E. coli*. This regulation relies on a physical interaction shown *in vivo* and *in vitro* between the C-terminal region of SpoT and the small protein YmgB. We show that YmgB can specifically and functionally promote SpoT-dependent accumulation of ppGpp *in vivo* (Figs. 1, 4, and 5). Moreover, despite strong sequence homologies and similar domain organizations, we did not detect a positive interaction between YmgB and RelA (Fig. 4) supporting a specific physiological role of YmgB on SpoT activities.

The C-terminal half of RSH enzymes is thought to play pivotal roles in sensing nutrient starvation and controlling the enzymatic state of the N-terminal half (8, 34, 35). In that sense, SpoT’s synthetase and hydrolase activities can be regulated by direct binding of heterologous partners to the C-Terminal region. The Acyl Carrier Protein (ACP) directly binds to the TGS domain of SpoT to favour (p)ppGpp synthesis over hydrolysis upon fatty acid starvation (18). Moreover, the nature of the fatty acid derivatives bound to ACP seems important to transduce the fatty acid status of the cell to SpoT (18). Similarly, to ACP, the anti-sigma factor Rsd can also interact with the TGS domain. However, and in contrast to ACP, the Rsd-SpoT interaction stimulates the hydrolase activity during carbon downshift (20). Importantly, only the dephosphorylated HPr (but not phosphorylated Hpr), a member of the phosphoenolpyruvate-dependent sugar phosphotransferase, was previously shown to bind Rsd in order to sequester it and therefore could antagonize this stimulatory effect (20). In addition, the ACT domain of SpoT is also needed to interact with the phosphorylated enzyme EIIA^Ntr^ to trigger (p)ppGpp accumulation in response to glutamine deprivation in *Caulobacter crescentus* (36, 37). Our observation that the TGS-Helical domains of SpoT (Fig. 6) are necessary and sufficient for YmgB binding further reinforces the pivotal role of the C-terminal half in controlling the reciprocal Hydrolase/Synthetase activities. However, unlike the other interacting partners mentioned above, the regulation of SpoT activities by YmgB does not rely on additional input (*e.g.* post-translational modification). Indeed, our results show that YmgB protein level is important for fine-tuning intracellular ppGpp level. Ectopic expression of *ymgB* is sufficient to trigger SpoT-dependent ppGpp accumulation in absence of nutritional stress (Fig. 1) and the SpoT-YmgB ratio seems to control the switch between SpoT activities (Fig. 3). Mechanistically, we observed that ppGpp accumulation upon YmgB induction also occurs in absence of RelA arguing that YmgB directly triggers ppGpp synthesis from SpoT (Fig. 1*B*). Moreover structural analysis on RelA/SpoT Homolog proteins suggest that theses enzymes avoid to simultaneously synthetize and degrade ppGpp, primarily to safeguard against futile cycle (7, 9, 33). Therefore, we propose that binding of YmgB to SpoT pushes the catalytic balance of SpoT toward ppGpp synthesis rather than hydrolysis. SpoT is a central protein that reponds to various stress signal (14, 15, 16, 17). How different types of nutrient starvation can use the same or independent signal transduction pathways to trigger SpoT-dependent (p)ppGpp accumulation remains unclear. The recent identification of several protein partners controlling SpoT activities (including YmgB) confirms that SpoT is at the center of a complex network. These observations favor a model in which a specific interacting partner may have evolved to respond to a specific environmental stress. Further analysis of the temporal dynamics of the interacting network under different stress conditions will help to resolve this open question.

It was proposed that YmgB could be a two-component system (TCS) connector that activates the Rcs phosphorelay (and therefore positively regulates capsule synthesis as well as many other genes, and negatively regulates motility) (22). TCS connectors are a class of proteins that modulate the output of TCS or more complex phosphorelay systems by providing additional signal inputs that are different from those perceived by the sensor kinase (38, 39). TCS connectors can interfere with the signal transduction by affecting phosphorylation, phosphotransfer, and dephosphorylation reactions. Therefore, YmgB seems to transduce a signal to both Rcs and SpoT. Importantly, we ensured that activation of both pathways are not interdependent. Indeed, overexpression of *ymgB* triggers SpoT-dependent ppGpp accumulation in a strain devoid of the Rcs response regulator RcsB (Figs. 2 and S2). Moreover, a single amino acid substitution (YmgB^R74G^) is sufficient to fully alleviate SpoT-dependent ppGpp accumulation without affecting the activation of the Rcs system (Figs. 4 and S4). Given that connectors often create regulatory links between independent signal transduction pathways (39), it is tempting to speculate that YmgB might couple the activation of the stringent response and the Rcs system in response to an unknown signal which is yet to be uncovered.

However, and despite extensive analysis we were not able yet to identify nutritional or environmental stress triggering YmgB-dependent ppGpp accumulation in *E*. *coli*. The regulation of the *ycgZ-ymgABC* operon is rather complex and seems to be induced in response to a large variety of stresses (22, 40, 41, 42, 43). Additional analyses are underway to explore in depth the regulation of this operon and to further address the exact relevance of the SpoT-YmgB interaction and it physiological role in controlling the stringent response in *E. coli*.

## Experimental procedures

### Bacterial strains, plasmids and media

Bacterial strains, plasmids, DNA oligonucleotides and media used in this study are listed in Tables S1–S4.

### Growth assay

The ability of *E. coli* cells to grow under specific conditions was tested as follows. Single colonies were inoculated into 5 ml of LB broth, supplemented with the appropriate antibiotic(s), and cultured at 37 °C until stationary phase (∼12 h). The cultures were centrifuged, washed, and serially diluted in PBS buffer, and then 5 μl of respective dilution were spotted on nutrient agar (NA), M9-glucose minimal medium or SMG plates containing the appropriate concentration of antibiotic(s) and inducer(s) as indicated. Plates were incubated at 37 °C overnight or ∼36 h depending on whether they were spotted on NA, SMG or minimal medium, respectively.

### *In vivo* (p)ppGpp measurement

The levels of (p)ppGpp in the cells after *ymgB* overexpression were determined as described previously (21). Cells of Δ*relA* mutant harboring pEG25-*ymgB* or pEG25*-6his-ymgB* and pEG25-*ymgB*^*R74G*^ or pEG25-*6his*-*ymgB*^*R74G*^ were grown overnight in MOPS minimal medium (44) supplemented with 0.2% glucose, 2 mM phosphate, and amino acids at 40 μg/ml. Cultures were diluted 100 times in the same medium with 0.4 mM phosphate and incubated at 37 °C with shaking. At an OD_600nm_ of ∼0.5, they were diluted to an OD_600 nm_ of 0.05, labelled with 150 μCi of ^32^P, and grew to an OD_600nm_ of ∼0.20. At this point 1 mM IPTG was added to induce expression of *ymgB*. Then 100 μl of ^32^P-labeled cell samples were taken at the indicated time and 40 μl of ice cold 21 M formic acid was added to stop the reactions. Samples were placed on ice for 20 min and then centrifuged at 4 °C for 20 min at 14,000*g* to pellet cell debris. Five microliters of each sample were loaded onto PEI-Cellulose thin layer chromatography (TLC) plates (Merck-Millipore) prior to ascending development with 1.5 M KH2PO4 solution (pH 3.4). Plates were revealed by PhosphoImaging (GE Healthcare) and analyzed using ImageQuant software (GE Healthcare). The amount of (p)ppGpp was normalized to the total amount of G nucleotides observed in each sample, the total G being the sum of GTP, ppGpp and pppGpp detected. For double expression assay the Δ*relA* Δ*spoT* Δ*ymgB* strain harboring pEG25-*spoT* and pBbS2K-*ymgB* or pEG25-*spoT* and pBbS2K-*ymgB*^*R74G*^ or pEG25-*spoT*^*L567P*^ and pBbS2K-*ymgB*^*R74G*^ were subjected to the same protocol. Overnight cultures were diluted 100 times in the same medium with 0.4 mM phosphate and incubated at 37°C with shaking. At an OD_600nm_ of ∼0.5, they were diluted to an OD_600nm_ of 0.05, labelled with 150 μCi of ^32^P, and expression of *spoT* and its variant was induced with 5 μM IPTG and cell were grown up to an OD_600 nm_ of ∼0.20. At this point 200 ng/ml aTc was added to induce expression of *ymgB*. Samples were collected at the indicated times and analysed as described above.

### Bacterial two-hybrid assay

*In vivo* protein-protein interactions were tested using a bacterial two-hybrid (BTH) assay (29). This method is based on the reconstitution of the adenylate cyclase from *B. pertussis* (29). Proteins of interest were fused to T18 and T25 of adenylate cyclase using pUT18c (or pUT18) and pKT25 (or pKNT25) plasmids. Co-transformed with the pairs of plasmids, single colonies of the *cya*-deficient strain BTH101 (29) were re-streaked and incubated overnight at 30 °C. Then, 2 ml of LB supplemented with ampicillin and kanamycin was inoculated with single colony and incubated at 30 °C with shaking for 8 h. 5 μl of undiluted cultures were then spotted on NA agar plates supplemented with X-Gal 40 μg/ml as a colour reporter for β-galactosidase and adenylate cyclase activities. For β-galactosidase experiments, the same inoculum has been used and β-galactosidase activity was determined as described by Miller with the used of the TECAN microplate reader to follow OD_600 nm_ and OD_420 nm_.

### Screening for loss of interaction by *ymgB* random mutagenesis

*ymgB* encoding sequence has been mutagenized randomly through low fidelity of GoTaq polymerase from pUT18c-*ymgB* plasmid using EJM135 and EJM137 oligonucleotides. After 30 PCR cycles, DNA was purified and diluted to 1/100,000 for another DNA amplification by PCR, these steps have been repeated two times. Purified DNA from the third PCR amplification were cloned into pUT18c plasmid. BTH101 cells harboring pKT25*-spoT* was then transformed with the resulting pUT18c-*ymgB* mutated plasmid library and screened on X-Gal plates. White colonies were selected and sequenced.

### Isolation of suppressive mutations that restore YmgB^R74G^ interaction by *spoT* random mutagenesis

*spoT* encoding sequence has been mutagenized randomly through low fidelity of GoTaq as described above from pKT25-*spoT* plasmid using EJM160 and EJM162 oligonucleotides. Purified DNA from the third PCR amplification was cloned into pKT25 plasmid. BTH101 cells harboring pUT18c*-ymgB*^*R74G*^ was then transformed with the resulting pKT25-*spoT* mutated plasmid library and screened on X-Gal plates. Blues colonies were selected and plasmids were sequenced.

### Protein expression and purification

BL21 (DE3) competent cells were transformed with the pEG25-*spoT*^*TGS-Helical-6His*^, pLic07-*His6*-*TRX*-*TEV*-*ymgB* or pLic07-*His6-TRX-TEV-ymgB*^*R74G*^ and plated on selective NA. For pEG25-*spoT*^*TGS-helical-6His*^ expression, several colonies were picked up and inoculated into 100 ml LB containing 100 μg/ml ampicillin. The cultures were grown with shaking at 30 °C overnight. Overnight cultures were diluted to 1/50 into 1L of TB media supplemented 100 mg/L of ampicillin and shaken at 30 °C until OD_600 nm_ ∼0.5, then cells were induced with 0.5 mM IPTG and incubated at 30 °C for 4 h. For YmgB and YmgB^R74G^ expression, several colonies were picked up and inoculated into 100 ml LB containing 50 μg/ml kanamycin. The cultures were grown with shaking at 30 °C overnight. Overnight cultures were diluted to 1/50 into 1 L of LB supplemented with 50 μg/ml kanamycin and shaken at 30 °C until OD_600 nm_ ∼0.5, then protein production was induced with 0.5 mM IPTG and incubated at 30 °C for 4 h.

Finally, cells were centrifuged at 9000*g* for 20 min at 4 °C. Dry cell pellets were stored at −80 °C.

For YmgB and YmgB^R74G^ purification, cells were suspended in lysis buffer (Tris-HCl 50 mM pH 8.0, NaCl 300 mM, EDTA 1 mM, lysozyme 0.5 mg/ml, 1 mM phenylmethylsulfonyl fluoride (PMSF), DNase 20 μg/ml and MgCl_2_ 20 mM). The mixture was incubated for 1 h at 4 °C with gentle shacking and then subjected to three cycle of French-press lysis steps. The soluble fraction was obtained by centrifugation for 30 min at 200,000*g*. Recombinant proteins were purified by ion metal affinity chromatography using a 5-mL nickel sepharose column on an ÄKTA pure 25 (GE healthcare) pre-equilibrated in Tris-HCl 50 mM pH8.0, NaCl 300 mM, 10 mM Imidazole (buffer A). After several washes in buffer A, 6-His tagged proteins were eluted in buffer A supplemented with 250 mM Imidazole final and immediately desalted using Hiprep 26/10 Desalting column pre-equilibrated with buffer A. The resulting desalted proteins were mixed with 0.2 mg/ml of TEV protease and incubated for 2 h at RT and then loaded onto a 5 ml nickel sepharose column pre-equilibrated in buffer A, which selectively retains the TEV, TRX and the uncleaved proteins and contaminants. Untagged YmgB was collected in the flow-through, concentrated on a Centricon (Millipore; cutoff of 3 kDa), and passed through a HiLoad 16/600 Superdex 200 column pre-equilibrated with 50 mM Tris-HCl pH 8.0, 500 mM NaCl, 500 mM KCl, 2 mM ß-Mercaptoethanol, Glycerol 2%. The purity of YmgB preparations was assessed by SDS–PAGE (Fig. S6, *B* and *C*).

For SpoT^TGS-Helical^ purification, cells were resuspended in lysis buffer composed of 50 mM Tris-HCl pH 8.0, 500 mM NaCl, 10 mM imidazole, 2 mM ß-Mercaptoethanol, 0.5% CHAPS, Glycerol 2%, 1 mM EDTA, 0.5 mg/ml lysozyme, 1 mM phenylmethylsulfonyl fluoride, 20 μg/ml DNase and 20 mM MgCl_2_. The mixture was incubated for 1 h at 4 °C with gentle shaking and then subjected to three cycles of French-press lysis steps. Pellet and soluble fractions were separated by centrifugation for 30 min at 200,000*g*. The soluble fraction containing SpoT^TGS-Helical^ proteins were loaded at room temperature onto a 5-mL nickel sepharose column using an ÄKTA pure 25 apparatus (GE healthcare) pre-equilibrated with equilibrium buffer 50 mM Tris-HCl pH 8.0, 500 mM NaCl, 10 mM imidazole, 2 mM ß-Mercaptoethanol, Glycerol 2% and the immobilized proteins were eluted in elution buffer 50 mM Tris-HCl pH 8.0, 500 mM NaCl, 500 mM imidazole, 2 mM ß-Mercaptoethanol, Glycerol 2%. Eluted proteins were immediately subjected to size exclusion chromatography (SEC) purification using a Superdex 200 increase 10/300 Gl column pre-equilibrated with 50 mM Tris-HCl pH 8.0, 500 mM NaCl, 500 mM KCl, 2 mM ß-Mercaptoethanol and Glycerol 2%.The purity of SpoT^TGS-Helical^ preparations was assessed by SDS–PAGE (Fig. 6*C*).

### Bio-layer interferometry

YmgB or YmgB^R74G^ were biotinylated using the EZ-Link NHS-PEG4-Biotin kit with 1:1 ratio (Perbio Science, France) at 4 °C. After 2 h, the excess of the biotin was removed by using a desalting column (Zeba Spin; Perbio Science). All BLI experiments were performed at 25 °C using the Blitz apparatus (Sartorius France S.A.S) with shaking at 2200 rpm. Streptavidin biosensor (Sartorius France S.A.S) were first hydrated 10 min with 0.350 ml of 50 mM Tris-HCl pH 8.0, 500 mM NaCl, 500 mM KCl, 2 mM ß-Mercaptoethanol, Glycerol 2% and then loaded with 2.5 μM of biotinylated YmgB in the same buffer. The biosensors were then incubated in interaction buffer [50 mM Tris-HCl (pH 8.0), 500 mM NaCl, 500 mM KCl, 2 mM β-mercaptoethanol, 2% glycerol, and BSA 1 mg/ml) for 90 s to avoid the non-specific binding SpoT^TGS-Helical^ to the streptavidin biosensors. To study the binding of YmgB or YmgB^R74G^ to SpoT^TGS-Helical^, increasing concentrations of SpoT^TGS-Helical^ (2–50 μM in interaction buffer) were used with the following steps; 30 s baseline, 120s association and 120 s dissociation. In all experiments, a reference subtraction of the SpoT^TGS-Helical^ on the uncoated biosensors for each concentration tested was performed. The dissociation constant (K_D_) was calculated using the GraphPad Prism 5.0 software on the basis of the steady state level responses, by plotting on x axis the different concentrations SpoT^TGS-Helical^ and on the y axis the corresponding response measured 10 s before the end of the association. For K_D_ calculation, a nonlinear regression fit for xy analysis was used and one site (specific binding) as a model [corresponding to the equation y=Bmax∗x/(KD+x)].

## Data availability

All data supporting the findings of this study are available within the paper and its Supplementary Information.

## Supporting information

This article contains supporting information.

## Conflict of interest

The authors declare that they have no conflicts of interest with the contents of this article.
