# Supplementary material for: The YmgB-SpoT interaction triggers the stringent response in Escherichia coli
Source: J Biol Chem. 2023 Nov 4;299(12):105429. doi: 10.1016/j.jbc.2023.105429 (PMC10704370; doi:10.1016/j.jbc.2023.105429)
Supplement: Supporting information [file mmc1.docx]

**Supporting Information to:**

**The YmgB–SpoT interaction triggers the stringent response in *Escherichia coli***

Paul Guiraud^1^, Elsa Germain^1^, Deborah Byrne^2^ and Etienne Maisonneuve^1^*

^1^Laboratoire de Chimie Bactérienne, Institut de Microbiologie de la Méditerranée, CNRS-Aix Marseille Univ (UMR7283), Marseille, France

^2^Protein Expression Facility, Institut de Microbiologie de la Méditerranée, CNRS-Aix Marseille Univ, Marseille, France

***e-mail:** [**emaisonneuve@imm.cnrs.fr**](mailto:emaisonneuve@imm.cnrs.fr)

Running Title: Control of bacterial stringent response by YmgB

Classification: Microbiology

Keywords: (p)ppGpp; Stringent Response; SpoT; YmgB, AriR, Bacterial stress response; *E. coli*

**List of the material included:** Supporting Tables 1,2,3,4. Supporting Figure legends 1,2,3,4,5,6. Supporting Figures 1,2,3,4,5,6

**Table S1: Strains used in this work**

| **Strain** | **Genotype** | **Source** |
| --- | --- | --- |
| BTH101 | F- cya-99 *araD139 galE15 galK16 rpsL*(StrR) *hsdR2 mcrA1 mcrB1 relA1* | [29] |
| BL21 (DE3) | E. coli B F– *ompT* *gal* | NEB |
| MG1655 | Wild-type *E.coli* |  |
| *ΔrelA* | MG1655 *relA*::FRT | P1 from KEIO collection. (Resistance cassette has been flipped out). |
| *ΔrelA ΔspoT* | MG1655 *relA251*::*aphA* *spoT207::cat* | Sequential P1 transduction from CF16939 in MG1655 |
| *ΔymgB* | MG1655 *ymgB*::FRT | P1 from KEIO collection (Resistance cassette has been flipped out). |
| *ΔrelA ΔymgB* | MG1655 *relA::FRT ymgB::FRT* | P1 from KEIO collection (Resistance cassette has been flipped out). |
| *ΔrcsB* | MG1655 *rcsB::FRT* | P1 from KEIO collection (Resistance cassette has been flipped out). |
| *ΔrelA ΔrcsB* | MG1655 *relA::FRT rcsB::FRT* | P1 from KEIO collection (Resistance cassette has been flipped out). |
| *ΔrelA ΔspoT ΔymgB* | MG1655 *relA::FRT ymgB::FRT spoT207::cat* | P1 from KEIO collection (Resistance cassette has been flipped out). |

**Table S2. Plasmids used in this work**

| **Plasmids** | **Genotype** | **Source and primers** |
| --- | --- | --- |
| pEG25 | pUC bla PT5-lac promoter, AmpR | [21] |
| pEG25-*relA* (sd4 ttg) | bla PT5-lac , *relA* , AmpR | [21] |
| pEG25-*ymgB* | bla PT5-lac , *ymgB* , AmpR | This work (pEJM14/15) |
| pEG25-*6his-ymgB* | bla PT5-lac , *6his-ymgB*, AmpR | This work (pEJM175/15) |
| pEG25-*ymgB^R74G^* | bla PT5-lac , *ymgB _R74G_* , AmpR | This work (pEJM14/15) |
| pEG25*-6his-ymgB^R74G^* | bla PT5-lac, *6his-ymgB^R74G^*, AmpR | This work (pEJM175/15) |
| pEG25*-spoT* (sd8 ttg) | bla PT5-lac, *spoT*, AmpR | (Germain et *al.* 2019) |
| pEG25*-spoT^L567P^* (sd8 ttg) | bla PT5-lac, *spoT^L567P^*, AmpR | This work (pEJM260/210) |
| pEG25*-spoT^S484G^* (sd8 ttg) | bla PT5-lac, *spoT^S484G^* , AmpR | This work (pEJM260/210) |
| pEG25-SpoT^TGS-Helical^-6his | bla PT5-lac *spoT^TGS-Helical^-6his* ,AmpR | This work (pEJM627/628) |
| pBbS2K | pBbS2K minus RFP selfligated |  |
| pBbS2K*-ymgB* | pBbS2K, *ymgB* | This work (pEJM14/15) |
| pBbS2K*-ymgB^R74G^* | pBbS2K, *ymgB^R74G^* | This work (pEJM14/15) |
| pLic07 | KmR | Biox tal |
| pLic07-*His6-TRX-TEV-ymgB* | *KmR, His6-TRX-TEV-ymgB* | This work (pEJM195/196) |
| pUT18c | AmpR ,used for fusion at C-terminal end of the T18 polypeptide | [29] |
| pUT18c-Zip | AmpR, with leucine zipper of GCN4 | [29] |
| pUT18c-*ymgB* | AmpR , *ymgB* | This work (pEJM135/137) |
| pUT18c-*ymgB^R74G^* | AmpR ,with *ymgB^R74G^* | This work (pEJM135/208) |
| pKT25 | Used for fusion at the C-terminal end of the T25 polypeptide, KmR | [29] |
| pKT25-Zip | with leucine zipper of GCN4, KmR | [29] |
| pKT25*-relA* | KmR , with *relA* full length | [21] |
| pKT25*-spoT* | KmR , with *spoT* full length | [21] |
| pKT25*-spoT^ΔACT^* | KmR , with *spoT* deleted for ACT domain | [21] |
| pKT25-*spoT^ΔACT-CC^* | KmR , with *spoT* deleted for ACT and CC domains | [21] |
| pKT25-*spoT^ΔACT-CC-Hel^* | KmR , with *spoT* deleted for ACT, CC and Helical domains | [21] |
| pKT25-*spoT^Nter^* | KmR, with *spoT* catalytic (HD + SYNTH) | [21] |
| pKT25*-spoT^HD^* | KmR, with *spoT* hydrolase (HD) | [21] |
| pKT25-*spoT^ΔHD^* | KmR , with *spoT* deletaed for HD | [21] |
| pKT25*-spoT^Cter^* | KmR , with *spoT* regulatory (TGS + Helical + CC + ACT) | [21] |
| pKT25*-spoT^ΔHD-Syn-TGS^* | KmR , with *spoT* deleted for HD, SYNTH and TGS domains | [21] |
| pKT25-*spoT^TGS+Helical^* | KmR , with TGS and Helical domains of *spoT* | This work (pEJM8/396) |
| pKT25*-spoT^L567P^* | KmR , with *spoT^L567P^* | This work (pEJM160/162 + 320/321) |
| pKT25*-spoT^S484G^* | KmR , with *spoT^S484G^* | This work (pEJM160/162 + 462/463) |
| pKT25*-ymgB* | KmR , with *ymgB* | This work (pEJM135/137) |
| pUT18*-ymgB* | AmpR , with *ymgB* | This work (pEJM135/136) |
| pKNT25-*spoT* | KmR , with *spoT* full length | This work (pEJM160/161) |

**Table S3. DNA oligonucleotides used in this work**

| **Primers** | **Sequence (From 5’ to 3’)** | **Restriction Enzyme** |
| --- | --- | --- |
| EJM8 | cccctctagaCCTGCTGGAGCTGCAACAGA | XbaI |
| EJM14 | ccccgaattcGTCGACTCAAGGAGGTTTTATAAATGCTTGAAGATACTACAATTC | EcoRI |
| EJM15 | ccccggatccTTACATATCATCAGCTGTGT | BamHI |
| EJM135 | cccTCTAGAaATGCTTGAAGATACTACAATT | XbaI |
| EJM136 | cccGGTACCaACATATCATCAGCTGTGTATC | KpnI |
| EJM137 | cccggtaccTTACATATCATCAGCTGTGTAT | KpnI |
| EJM160 | ccctctagaaaTGTATCTGTTTGAAAGCCTGA | XbaI |
| EJM161 | cccGGTACCaAATTTCGGTTTCGGGTGACTTT | KpnI |
| EJM162 | cccggtaccTTAATTTCGGTTTCGGGTGAC | KpnI |
| EJM175 | ccccgaattcGTCGACTCAAGGAGGTTTTATAAATGcatcaccatcaccatcacATGCTTGAAGATACTACAAT | EcoRI |
| EJM195 | CCGAGAACCTGTACTTCCAATCAATGCTTGAAGATACTACAATTC |  |
| EJM196 | CGGAGCTCGAATTCGGATCCTTATTACATATCATCAGCTGTGTATCGC |  |
| EJM208 | ccccggtaccTTACATATCATCAGCTGTGTATCGCAACACGATTTCCAGTGTTTTTCCAATCACATCAGCTTT | KpnI |
| EJM210 | CCCCGGATCCTTAATTTCGGTTTCGGGTGAC | BamHI |
| EJM260 | cccgaattcGTCGACTAAAGGAAAAAAAAATTGTATCTGTTTGAAAGCCTGA | EcoRI |
| EJM320 | GTGCCGATGGCGTGCCGATCACCTTTGCGAA |  |
| EJM321 | TTCGCAAAGGTGATCGGCACGCCATCGGCAC |  |
| EJM396 | cccggtaccTTATTGCAGATTTTTCGCGACCACC | KpnI |
| EJM462 | CGTGATGATTCTGTAGGCCTGGGCCGTCGTC |  |
| EJM463 | GACGACGGCCCAGGCCTACAGAATCATCACG |  |
| EJM627 | ccccgaattcgtcgactcaaggaggttttataaATGTACGTTTTCACACCG | EcoRI |
| EJM628 | ccccggatccTTAgtgatggtgatggtgatgTTGCAGATTTTTCGC | BamHI |

**Table S4. Media used in this work**

| **Compounds** | **Quantities** |
| --- | --- |
| **Luria-Bertani (LB) broth** |  |
| Tryptone (Oxoid, LP0042B) | 10 g/L |
| Yeast extract (Oxoid LP0021B) | 5 g/L |
| NaCl | 10 g/L |
| **M9-glucose minimal medium** |  |
| Na_2_HPO_4_ | 60 mM |
| KH_2_PO_4_ | 22 mM |
| NaCl | 8 mM |
| NH_4_Cl | 20 mM |
| MgSO_4_ | 1 mM |
| CaCl_2_ | 100 µM |
| Thiamine | 1 µg/mL |
| Glucose | 0.2% |
| Agar bacteriological (Oxoid, LP0011) | 15 g/L |
| **Nutrient agar (NA)** |  |
| Nutrient agar (Oxoid, CM0003B)  (Lab-Lemco’ Powder 1g/L; Yeast extract 2g/L; Peptone 5g/L; NaCl 5g/L; Agar 15g/L) | 28 g/L |
| **SMG (M9-glucose minimal medium supplemented with** |  |
| L-Serine | 40 µg/mL |
| L-Methionine | 40 µg/mL |
| Glycine | 40 µg/mL |
| Agar bacteriological (Oxoid, LP0011) | 15 g/L |
| **Terrific broth (TB)** |  |
| Peptone | 1.2% |
| Yeast extract | 2.4% |
| K_2_HPO_4_ | 72 mM |
| KH_2_PO_4_ | 17 mM |
| Glycerol | 0.4% |

**Supplementary Figure Legends**

**Figure S1 related to Figure 1: YmgB stimulates SpoT dependent ppGpp accumulation.**

(**A**) The WT and Δ*relA* mutant were transformed with pEG25 (Ø) or pEG25 harboring either *relA, ymgB* under an IPTG-inducible promoter. Serial dilutions of stationary-phase cultures were spotted on nutrient agar (NA) and SMG plates with the indicated concentration of IPTG. The results are representative of three independent experiments with similar results.

(**B**) Growth curve of Δ*relA* strain transformed with pEG25 harboring *ymgB* in SMG liquid medium. Overnight culture in liquid M9 medium were 100 times diluted in liquid SMG medium supplemented with different concentration of IPTG (0, 25, 50 or 100µM) and growth was monitored at 600nm by using TECAN microplate reader. (**C**) The WT and the (p)ppGpp^0^ (Δ*relA* Δ*spoT*) strain were transformed with pEG25 (Ø) or pEG25 harboring either *relA* or *ymgB* under an IPTG-inducible promoter. Serial dilutions of stationary-phase cultures were spotted both on NA and minimal medium (M9) plates supplemented with the indicated concentration of IPTG. The results are representative of three independent experiments with similar results.

**Figure S2 related to Figure 2: SpoT-dependent accumulation of ppGpp is independent of RcsB.**

The WT, Δ*rcsB,* Δ*relA* and Δ*relA* Δ*rcsB* strains were transformed with pEG25 (Ø) or pEG25 harboring *relA* or *ymgB,* under an IPTG-inducible promoter. Serial dilutions of stationary-phase cultures were spotted NA and SMG plates supplemented with the indicated concentration of IPTG. This experiment was repeated three times with similar results.

**Figure S3 related to Figure 3: The YmgB/SpoT ratio determines growth on SMG plates.**

The WT strain was co-transformed with pEG25 (Ø) or pEG25 harboring *spoT* gene under an IPTG inducible promoter and with pBbS2K (Ø) or pBbS2K harboring either *ymgB* or *ymgB^R74G^* gene under an anhydrotetracycline (aTc) promoter. Serial dilutions of stationary-phase cultures were spotted both on SMG and NA with gradual concentration of IPTG (to induce *spoT*) and aTc (to induce *ymgB*). Experiments have been repeated three times with similar results.

**Figure S4 related to Figure 4: Analysis of YmgB-SpoT interaction, variants functionality and protein level.**

(**A**) Bacterial two hybrid assay with YmgB or YmgB^R74G^ and SpoT and SpoT variants. The results shown for the pUT18C*ymgB^R74G^*/pKT25*spoT^L567P^* and pUT18C*ymgB^R74G^*/pKT25*spoT^S484G^* interaction are reused images from source image shown in Figure 5B. Similarly, the results shown for the pUT18C*ymgB*/pKT25*spoT^TGS-Helical^* interaction is a reused image from source image shown in Figure S6A. (**B**) Bacterial two hybrid between YmgB and SpoT in different combinations. BTH101 cells were co-transformed with plasmids harboring pUT18c and pKT25 fusions were spotted on X-Gal agar base plates (see Materials and Methods). (**C**) *ymgB^R74G^* overexpression does not suppress the non-growing phenotype of the Δ*relA* mutant on SMG plates. The Δ*relA* mutant was transformed with pEG25 (Ø) or pEG25 harboring *ymgB* or *ymgB^R74G^* under an IPTG-inducible promoter. Serial dilutions of stationary-phase cultures were spotted on NA and SMG plates with 100 µM of IPTG. The results are representative of three independent experiments with similar results. (**D**) *In vivo* (p)ppGpp accumulation following ectopic expression of *6his-ymgB* and *6his-ymgB^R74G^*. The Δ*relA* mutant carrying either *6his-ymgB* or *6his-ymgB^R74G^* on pEG25 was grown exponentially in phosphate MOPS minimal medium (see material and methods). Samples were collected before and after *ymgB* induction (1mM IPTG) prior to nucleotide extraction and separation by TLC. The autoradiogram is representative of three independent experiments. (**E**) Protein level of 6His-YmgB and 6His-YmgB^R74G^ when produced in WT and Δ*relA* strains. Cells were grown exponentially at 37°C in LB (OD_600_ 0.5), then cells were induced with 200μM of IPTG for 1h. Samples were prepared and equal amount of total proteins were loaded on SDS-PAGE. Immunoblot analysis were performed with antibody Penta-His-HRP conjugated diluted 1/10.000 (GeneTex) (upper panel). Loading control (lower panel) were performed with a primary α-EF-Tu antibody diluted 1/20.000 (Hycult Biotech) and a secondary anti-mouse antibody diluted 1/10.000 (Sigma). Visualizations were performed using luminata crescendo HRP substrate (Millipore) and ImageQuant Las4000 (GE Healthcare). (**F**) YmgB^R74G^ is correctly induced and stimulates Rcs phosphorelay as shown by mucoid phenotype on SMG plates. WT strain was transformed with pEG25, empty (Ø) or harboring either *ymgB* or *ymgB^R74G^* under an IPTG-inducible promoter. Cells were serial diluted and spotted on SMG plate with 50µM of IPTG. This experiment was repeated three times with similar results.

**Figure S5 related to Figure 5: *ymgB^R74G^* promotes growth on SMG when co-expressed *spoT^L567P^***

(**A**) The Δ*relA* Δ*spoT* Δ*ymgB* strain was co-transformed with pEG25 empty (Ø) or harboring *spoT,* *spoT^L567P^ or spoT^S484G^* gene under an IPTG-inducible promoter and with pBbS2K empty (Ø) or harboring *ymgB* or *ymgB^R74G^* gene under an anhydrotetracyclin (aTc) promoter. Cells were serially diluted and spotted on NA or SMG medium with 25µM of IPTG (to induce *spoT*) and 100 ng/mL of aTc (to induce *ymgB*). Experiments have been repeated three times with similar results. (**B**) The Δ*relA* Δ*spoT* Δ*ymgB* strain co-transformed with pEG25 harboring *spoT^L567P^* gene under an IPTG-inducible promoter and with pBbS2K empty (Ø) or harboring *ymgB^R74G^* gene under an anhydrotetracyclin (aTc) promoter were grown exponentially in phosphate MOPS minimal medium with 10 µM IPTG. Samples were collected before and after *ymgB* induction (500 ng/mL aTc) prior to nucleotide extraction and separation by TLC. The autoradiogram is representative of three independent experiments.

**Figure S6 related to Figure 6: YmgB can interact with the TGS-Helical domains of SpoT**

(**A**) *E. coli* BTH101 cells were co-transformed with plasmid derivatives pUT18c-*ymgB* and pKT25 with the full-length or truncated *spoT* gene as indicated. Stationary-phase cultures were spotted on NA plates containing X-Gal as a blue color reporter for positive interaction. The bars showing β-galactosidase activity are represented as the means of three independent experiments, the error bars depict the SDs.

Purification of YmgB (**B**), YmgB^R74G^ (**C**) and SpoT^TGS-Helical-6His^ (**D**) using size-exclusion chromatography (SEC). The elution volume (from a HiLoad 16/600 Superdex 200 column) is plotted on the x axis, and the 280-nm absorbance on the y axis. The SEC elution fractions showed by black arrows were analyzed by 12% SDS-PAGE. The name of the analyzed protein is indicated on the right. For SDS-PAGE molecular weight markers (in kDa) are indicated on the left.

**Figure S7: Prediction of the YmgB-SpoT complex by AlphaFold2.**

(**A**) Modeling was done using Alphafold 2.3.1. In total 10 predictions were generated with a final relaxation step. The predictions were ranked according to IPTM+PTM and the top prediction consistent with constraints based on our *in vivo* results (Fig 4 and 6) was chosen (ranked 3). Top left: predicted YmgB structure in magenta. The surface exposed residues important for the YmgB SpoT-interaction are colored in red based on results shown in Figure 4C. Top right: predicted SpoT structure. the hydrolase domain is represented in marine blue, the synthase domain in salmon, the TGS domain in yellow, the Helical domain in pale green, the CC domain in orange and the ACT domain in cyan. Residue which when mutated restore the interaction with the YmgB^R74G^ variant are colored in green. Bottom left: predicted YmgB-SpoT interaction. SpoT is represented in transparent. Bottom right: predicted YmgB-SpoT interaction with a y rotation of 120°. YmgB is represented in transparent. (**B**) AlphaFold2 confident score (predicted LDDT per position) mapped on the model.

**
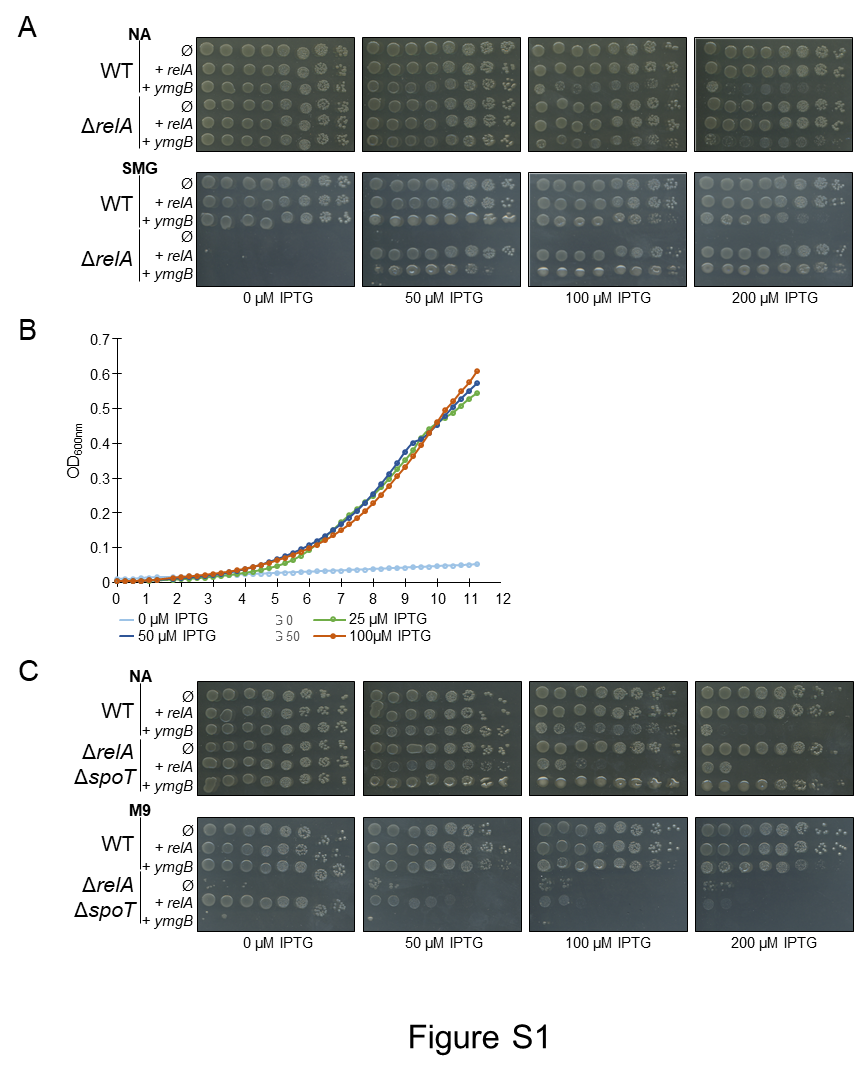
**

**Figure S1**


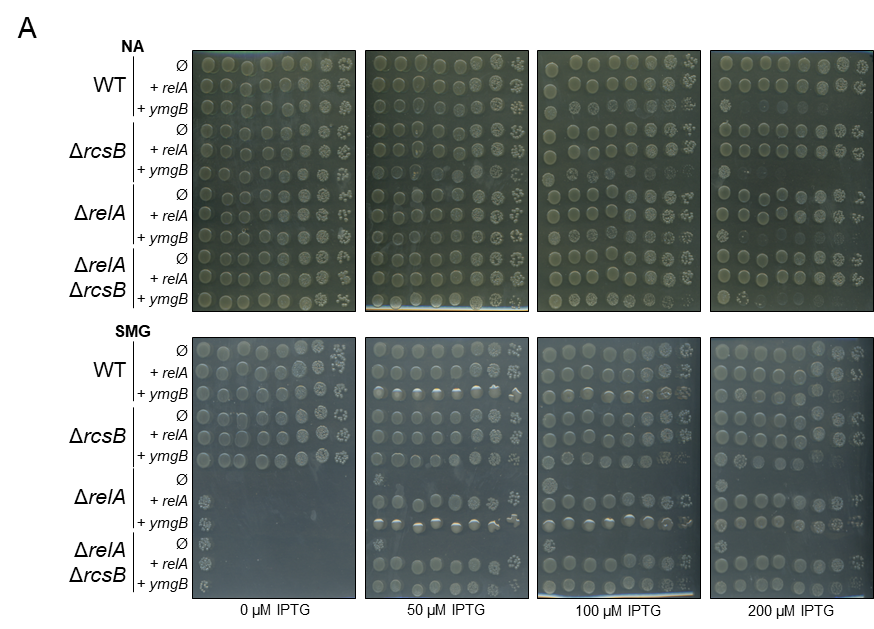


**Figure S2**


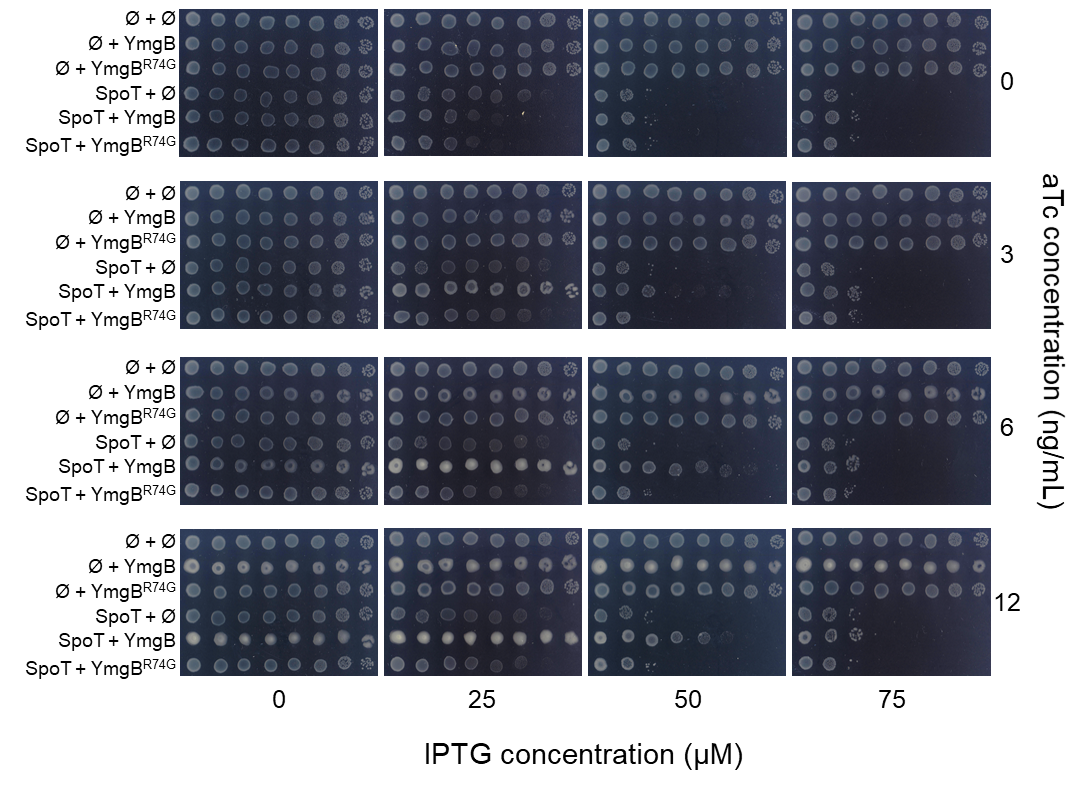


**Figure S3**

**
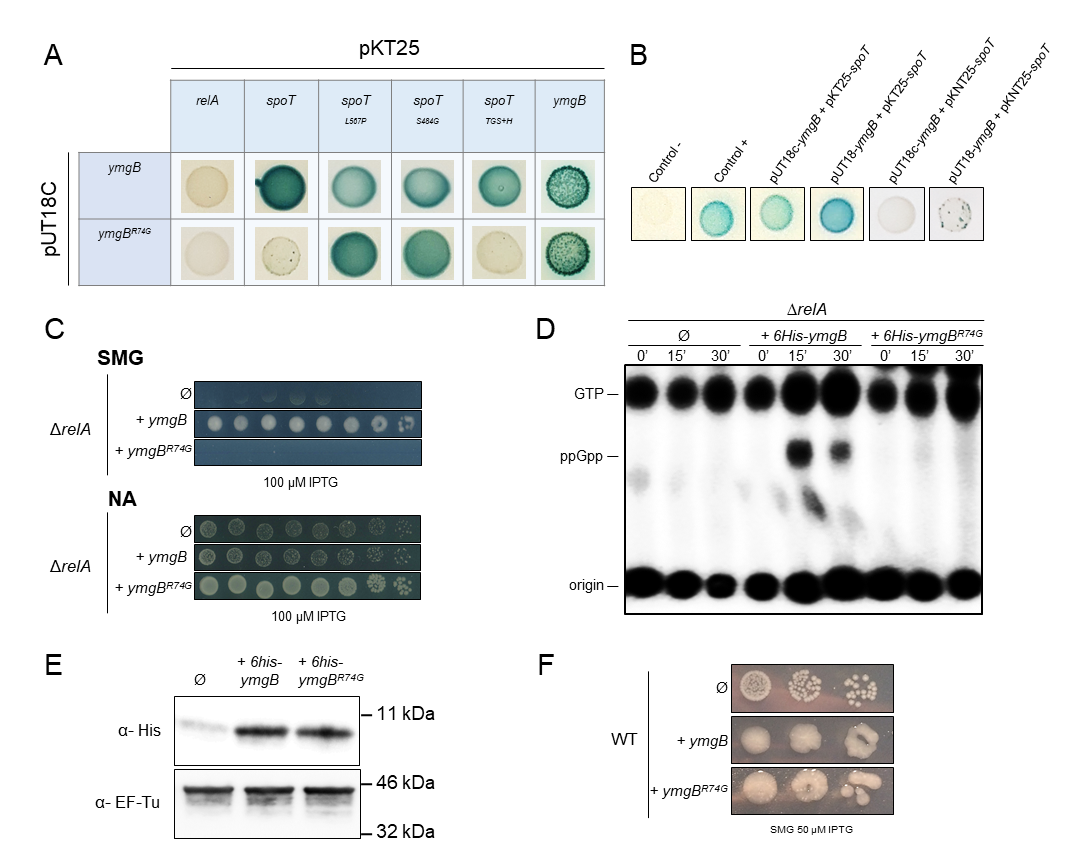
**

**Figure S4**

**
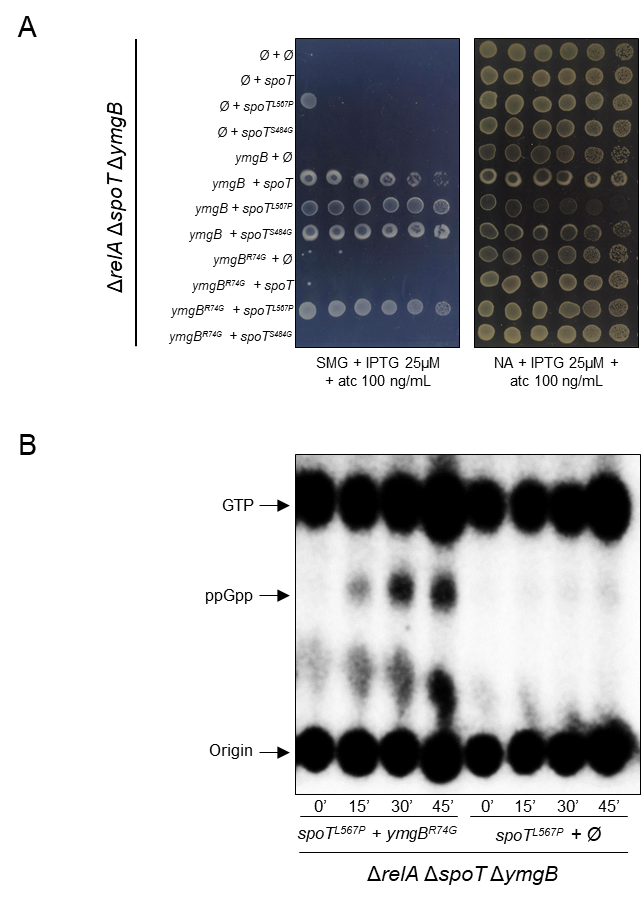
**

**Figure S5**

**
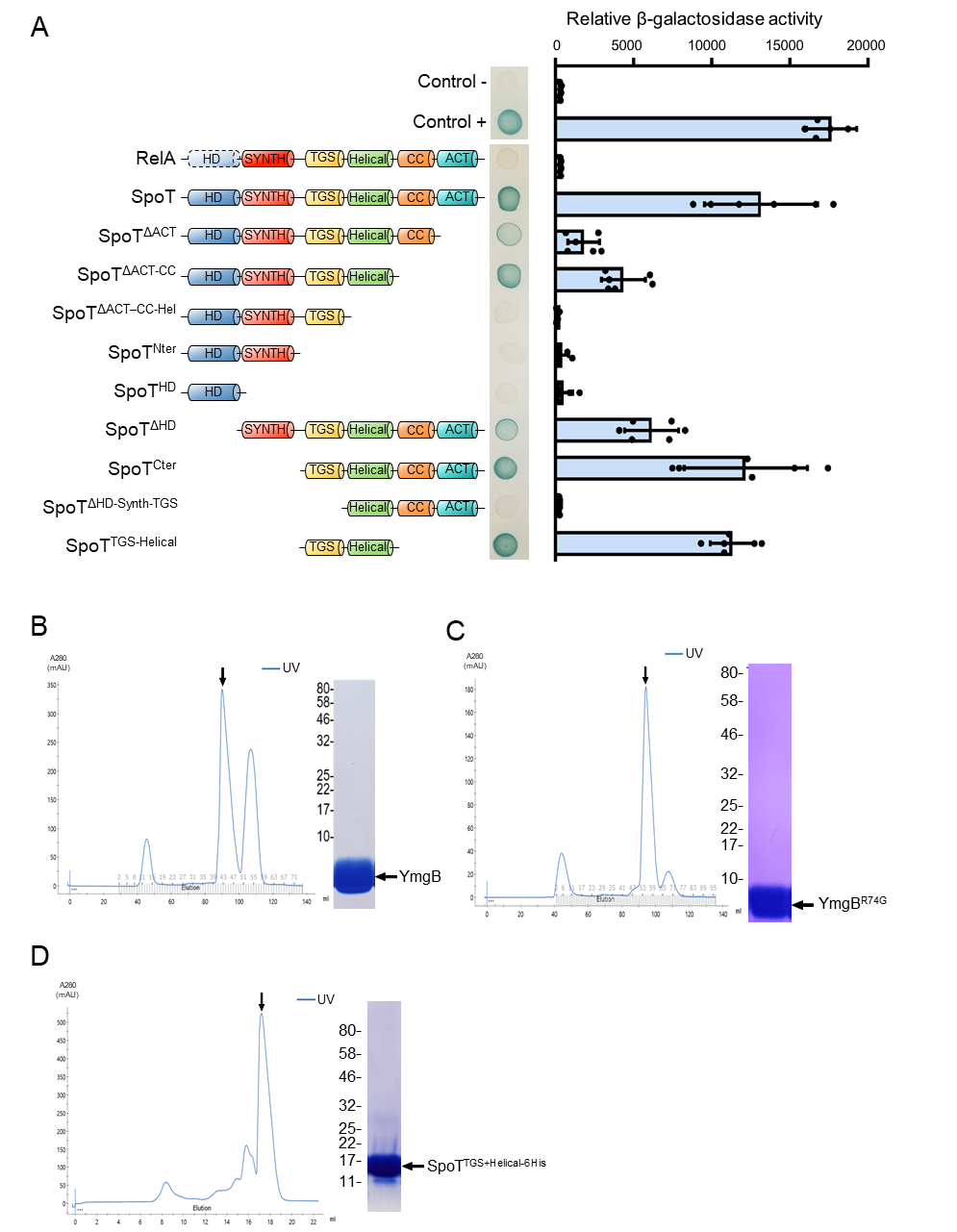
**

**Figure S6**

**
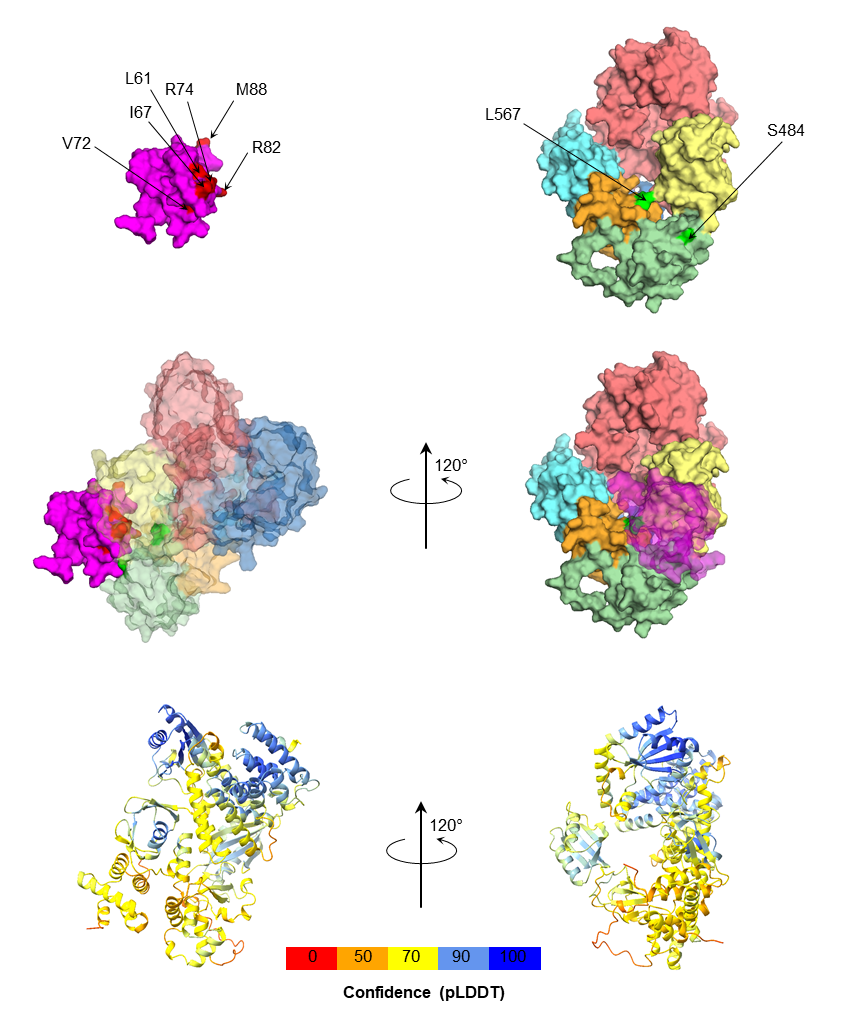
**

**Figure S7**
